# Supplementary material for: Patterns of Intron Gain and Loss in Fungi
Source: PLoS Biol. 2004 Nov 30;2(12):e422. doi: 10.1371/journal.pbio.0020422 (PMC532390; doi:10.1371/journal.pbio.0020422)
Supplement: Table S1 — Also available at http://genes.mit.edu/NielsenEtAl/. (4.3 MB ZIP). [file pbio.0020422.st001.zip › NielsenEtAl/html/220.html]

AN4556.1.NCU00080.1.MG10154.1.FG06717.1


```
 CLUSTAL W (1.82) Multiple Sequence Alignments - Introns Inserted


Sequence 1: NCU00080.1	362 aa
Sequence 2: MG10154.1	356 aa
Sequence 3: FG06717.1	355 aa
Sequence 4: AN4556.1	359 aa
Alignment Length: 376 aa
Number Identitical Residues: 116 aa
Alignment Score (without introns) 7040


MG10154.1 	------MALPALNALEECSDFSKTVEPFLPQLYELPNKLAEVFTSGGS--LRALYLGTNP
NCU00080.1	MILPYATALPKLGALQDCSDFSKTVTPFLPQLYALPRQTLDTIFNGGS--FLKLYMETNP
FG06717.1 	------MALPYLKSIEDCGEYARAVEPYIPQLYALPRQFLDNVASPDG--LRQLYVDTNP
AN4556.1  	----MTLPLPDVESLADCVSFNHAVRPFLSQVAALPERLQPAIAAKDVNALKDIYLSTNP
          	     : .** : :: :* .: ::* *::.*:  **.:    .   . .::  :*: ***

MG10154.1 	LISAFAFSLVLGFVFLVVSEINKNYSQVDRCWSLLPTVYLAHFDVWARMAGLPSERLDTA
NCU00080.1	FISGLGISILLGGVFLVAAEVNRNYSQVDRFWSILPTLYIAHFATWARLAGIPSRRIDAA
FG06717.1 	LISGFAISVALTVPFLVVSEINRNYSQVDRMWSILPNLYVVHLSIWARLAGVSSSRIDLV
AN4556.1  	LATALAFTIFLSVLFIVFSEINRNYSQVDRFWSILPSVFNVHFAVWARLSGLRTLNLDTI
          	: :.:.::: *   *:* :*:*:******* **:**.:: .*:  ***::*: : .:*  

MG10154.1 	LFFSTVWS0IRLTYNYARKGGYNVGSEDYRW2EIVRKNAP-AWAFHLLNVVFISFIQSIL
NCU00080.1	LLFSTIWS0IRLTYNYWRKGGYNVGSQDYRW~DIVRASMP-KLAFHILNWTFISFIQSVL
FG06717.1 	SLATTLWS0CRLTYNYWRKGGYEKGSEDYRW2AILQKYVP-RPVWFIFNVTFISFYQSVL
AN4556.1  	AAISVIWS0VRLTFNYWRRGGYSIGSEDYRW~SIIRSRVNNRFAFFLFNITFISVIQPLL
          	   :.:**  ***:** *:***. **:****  *::    .  .:.::* .***. *.:*

MG10154.1 	LF-LIAAPAYPMLLAIQF--EPQASTADYVFMSIQLGLVALEWFADQQQWD~FHGAKKQY
NCU00080.1	LY-MLASPVYVLLLATQF--EPELSTADIGFVVMELGLILTEFIADHQQWV1FQSAKIEY
FG06717.1 	LFSFSCVPAYAILLSTKF--EENVTPADILFFLTMVGLIYSEWVSDGQQWD1FHAAKHKY
AN4556.1  	LL-LLTTPTYNFLLLARLPGGEAFELPDLIFSRVALVFIILEFFADQQQWR~FQNAKHEY
          	*  :   *.* :**  ::..      .*  *    : ::  *:.:* ***  *: ** :*

MG10154.1 	QESAKVP----QGYTHAEVEQGFVSSGLWAYSRHPNFAAEQTIWFLLYQWSCYDTKVLYS
NCU00080.1	KTSGQIP----AGHKQADLDRGFITSGLWAYSRHPNFAAEQSIWLTLYQWGCFATNTLYN
FG06717.1 	QAEAKVPR--EFSYTQADLDRGFNTSGLWAYSRHPNFAAEQLVWFVLYQWSCYATKNLYS
AN4556.1  	NTSARIPGNLKDQYDPEDLERGFVVSGLWSLSRHPNFAAEQAIWLTLYLWNAYRTEHYVQ
          	: ..::* . .  :   ::::**  ****: ********** :*: ** *..: *:   .

MG10154.1 	WAAVGSVSLLSLFQGSTWLTELITSGKYPDYKDYQKQVGMFVP-TGPAYQARAISKKPKI
NCU00080.1	WTIVSPIMLMSVFQGSTWLTERITAGKYPEYSAYQKQVGTFVPKTLKGYKT----PVPKF
FG06717.1 	YTLAGSAALIMLFQGSTWLTELITVGKYSEYPKYQKQVGMFLPKSLQPYKT----PPPKV
AN4556.1  	WTALGVIGLMAIFQGSVRLTEEISARKYPEYQEYQARVGRFIPRLSIT---------PRY
          	::  .   *: :****. *** *:  **.:*  ** :** *:*              *: 

MG10154.1 	IKTSELAKKNEKKKHK---
NCU00080.1	IRTSDLAKKLQEKENKKQK
FG06717.1 	IRTSDLAKNESKKEA----
AN4556.1  	KGNKNKKRVAHGKKEE---
          	  ..:  :    *: .
```
